# Reproductive Biology and Feeding Ecology of The Blue Swimming Crab (*Portunus pelagicus*) in Northern Coastal Waters, Sri Lanka

**DOI:** 10.21315/tlsr2022.33.2.8

**Published:** 2022-07-15

**Authors:** SSK Haputhantri, KHK Bandaranayake, MIG Rathnasuriya, KGS Nirbadha, SJWWMMP Weerasekera, AASH Athukoorala, RAM Jayathilaka, HACC Perera, S Creech

**Affiliations:** 1 Marine Biological Resources Division, National Aquatic Resources Research and Development Agency (NARA), Colombo 15, Sri Lanka; 2 Pelagikos Private Limited, 16 Welikadawatte Road, Rajagiriya 10107, Sri Lanka; 3 Faculty of Fisheries and Ocean Science, Ocean University of Sri Lanka, Mahawela Road, Tangalle, Sri Lanka; 4 Department of Zoology and Environmental Management, Faculty of Science, University of Kelaniya, Kandy Road, Kelaniya, Sri Lanka

**Keywords:** *Portunus pelagicus*, Spawning, Maturity, Gonadosomatic Index, Fecundity, Feeding

## Abstract

Blue swimming crab (*Portunus pelagicus*) fishery has emerged to become an important export-oriented fishery in Sri Lanka over a decade and recently resulted in a rapid increase in the exploitation. The present study attempts to understand the reproductive biology and feeding ecology of blue swimming crab which will be vital in the management of capture fishery. Five major landing sites in the Jaffna district in Northern Sri Lanka, where blue swimming crab is frequently landed throughout the year were selected for the study. Biological parameters relating to sex, carapace width, body weight, maturity, and egg sac colour with egg sac weight were recorded at the field from November 2014 to October 2015. Randomly selected crab samples were brought to the laboratory and analysed for their maturity stages, length at first maturity, Gonadosomatic Index, fecundity, and gut contents. The study revealed that male crabs mature at a smaller size than females. The sex ratio varied greatly with time and males were always dominant in the catch. The blue swimming crabs in the Northern waters of Sri Lanka spawn throughout the year, with two spawning peaks in February and May. The total fecundity of ovigerous blue swimming crab increased with increased carapace width and body weight and it ranged from 123,482 to 3,179,928 eggs, with an average of 884,982 ± 676,420. A remarkably higher percentage of empty stomachs were observed under the present study in both mature and immature crabs and this could be due to lack of food availability in the environment and different digestibility rates of food items. The diet of blue swimming crab is highly variable reflecting the ability to adopt to different modes of feeding.

**Highlights**

- Fecundity of *P. pelagicus* in the Northern waters of Sri Lanka is comparatively higher than the fecundity of the same species reported in Western Australia and Southern Thailand.
- Male crabs mature at a smaller size than females.
- The presence of sand particles and fishing net material in the stomach of immature crabs in a higher percentage was evidence for their non-selective food habit nature.

## INTRODUCTION

The blue swimming crab, *Portunus pelagicus* has a wide geographical distribution and is an important commercial species throughout the tropical and subtropical waters (Zainal 2013; Kunsook *et al*. 2014). The species is usually found in large numbers in shallow bays with sandy bottoms (Williams 1981). The geographical extent of the blue swimming crab in Sri Lanka extends from Chilaw on the Northwest coast to Trincomalee on the Northeast coast. However, the resource is more abundant in the shallow coastal waters of the Palk Bay which is bounded by three administrative districts in the Northern province: Mannar, Kilinochchi and Jaffna. A year-round fishery exists in the above areas with peak fishing seasons which is mainly controlled by the monsoonal weather.

Fishing is conducted in the coastal waters by outboard engine motorised and non-motorised fishing crafts. These boats are operated with gillnets (either monofilament or nylon) and the widely used mesh sizes are 8.9 cm and 11.4 cm. Apart from that, a substantial quantity of *P. pelagicus* is caught as a non-target species by the bottom trawlers operated mostly targeting shrimps and for fyke nets and stake nets.

After the end of the civil conflict in 2009, the export oriented blue swimming crab fishery in Northern waters of Sri Lanka has emerged as an increasingly important fishery because of its economic value. A rapid growth in the *P. pelagicus* annual catch as well as in the exports could be observed after the conflict. A quite high fishing pressure on the resources has been directed during this period and resources are being heavily exploited at present. The global demand for *P. pelagicus* is the reason for massive exploitation. Since sustainable exploitation of the resource is key, a great concern has been drawn now for research and resource management. The Seafood Exporters’ Association of Sri Lanka (SEASL) laid the foundation in 2013 for the Sri Lanka blue swimming crab Fishery Improvement Project (FIP) with the aim of creating and implementing a local plan that will improve the economic, social and ecological sustainability of the fishery. According to the plan of FIP, it has emphasised the importance of conducting scientific research and assessments on *P. pelagicus* with the aim of providing necessary scientific advice for the management of the blue swimming crab fishery in view of sustainable utilisation. A study on reproductive biology, especially spawning period and fecundity, is important to have a full understanding of the population dynamics. Basic information related to population dynamics and biology of *P. pelagicus* like species is vital for policy makers and managers to establish regulation measures for sustainable utilisation of the resource (Safaie *et al*. 2012). The determination of the sex ratio and of the sequence of changes in maturity stages during the year is of considerable importance in building a thorough knowledge of the general biology of an exploited stock (Holden & Raitt 1974). Moreover, knowledge of the fecundity of a species is also an important factor and it is used to calculate the reproductive potential of a stock (Holden & Raitt 1974). Studies on feeding ecology also play an important role due to its relationship to various trophic levels in the food web (Kunsook *et al*. 2014).

An understanding of the reproductive biology of *P. pelagicus* is fundamental to provide sound scientific advice for future management of the fishery. On the other hand, studying the feeding ecology of *P. pelagicus* may be important for blue swimming crab farming. The study was undertaken to improve the knowledge of *P. pelagicus* reproduction in order for better management of the wild stock. The food and feeding aspects of *P. pelagicus* explored under the study could probably be made use of for blue swimming crab farming. Jaffna district was selected for this investigation since *P. pelagicus* resource is rich in Jaffna and a year-round fishery exists there.

## MATERIALS AND METHODS

### Training of Field Staff

Prior to beginning of data collection in Jaffna, enumerators employed for biological sampling went through a training on the identification of *P. pelagicus*, their sex and maturity determination, taking length and weight measurements and filling data sheets etc. Accordingly, they have first undergone for an inhouse training followed by a training at the crab landing sites in the Jaffna district in October 2014 and the whole training programme was conducted by three scientists attached to Marine Biological Resources Division (MBRD) of National Aquatic Resources Research and Development Agency (NARA).

### Biological Data Collection

**Figure 1:**
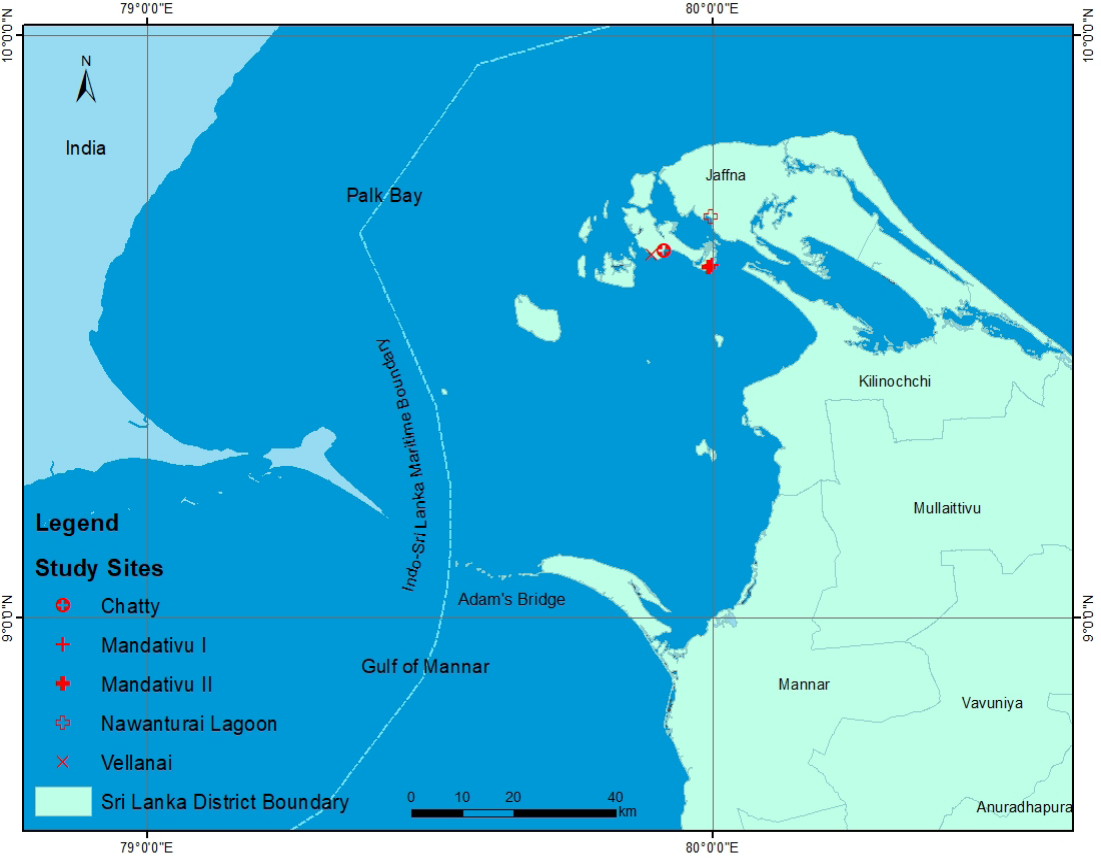
Blue swimming crab sampling sites in Northern coastal waters, Sri Lanka.

The preliminary field visit was made to Jaffna district in September 2014 to select the best sampling locations for biological sampling. Biological data collection was carried out at the landing sites in Mandativu (two landing sites), Vellanai (one landing site), Chatty (one landing site) and Nawanthurai lagoon (one landing site) in Jaffna district during November 2014–October 2015 (Fig. 1). Eight enumerators (two enumerators per site) were assigned for the first four landing sites for biological data collection of *P. pelagicus* whereas a research team of MBRD comprised of two Scientists and one Research Assistant visited monthly to Nawanthurai lagoon where juvenile crabs were mostly landed as a non-target species for prawn stake net fishery in order to obtain biological data of *P. pelagicus*. Biological data collection was carried out for five consecutive days per month mostly in the second week of the month. A well-trained field coordinator was also recruited for coordinating the data collection activities carried out by enumerators. The carapace width and weight with sex and maturity status: mature/ immature of *P. pelagicus* landed by the fishing crafts were randomly measured and recorded. Around 150 individual crabs were measured per day for a site. Whole data collection process was closely supervised by the above research team of MBRD.

### Laboratory Analysis of Biological Samples

In each month, freshly caught *P. pelagicus* samples obtained from the sampling sites were brought to the Marine Biology Laboratory of MBRD with ice and were properly preserved in a deep freezer until biological analysis was carried out. Each crab was examined and measured for the following: total length (mm), carapace width (mm), body weight (g), gonad weight (g), sex, maturity stages, fecundity, stomach weight (g) and stomach fullness.

### Maturity Stages and Spawning Season(s)

The carapaces of *P. pelagicus* were opened and the ovaries were examined for their stage of maturity. Maturity stages of females were classified into six categories following the procedure adopted by Dineshbabu *et al*. (2007). The categorisation of ovarian development based on size and colour were as follows:

1. F1: Immature, Gonad immature, ovary very thin and transparent (colourless)
2. F2: Early maturing, Gonad maturing, ovary changed colour to creamy, but not extending into hepatic region
3. F3: Late maturing, ovary became enlarged and changed colour to yellow, extending some 1/3–1/4 of the hepatic region
4. F4: Gonad mature, the ovary covered most part of hepatic region, and turned orange or reddish orange
5. F5: Berried females, Matured females externally carrying eggs (ovigerous female) egg sacs
6. F6: Spent, the empty flat pinkish gonads with a coffee brown abdomen

The ovary was then removed and weighed. The Gonado-Somatic Index (GSI) was calculated:


$$\text{GSI}=\frac{\text{Gonad weight}}{\left(\text{Body weight}-\text{gonad weight}\right)}\times 100$$

Spawning season was determined from the percentage of ovigerous females and GSI value in each month.

Male gonad maturity stages were classified into two categories: M1 = immature and M2 = mature.

### Fecundity

Fecundity was calculated as the number of eggs carried externally by the female (Kumar *et al*. 2000). Yellow/orange egg sac, the frequently found stage was analysed to determine the fecundity (Johnson *et al*. 2010). Egg-bearing pleopods were removed carefully; the wet weight of the whole egg mass was measured to the nearest 0.01 g using an electronic balance. Corresponding carapace width to the egg sac mass was also recorded. Approximately 0.1 g sub samples were taken from three different places of egg sac and the number of eggs in each sub sample was counted to get the average value. The total fecundity (TF) was estimated using the following equation:


$$TF=\frac{w}{W}*OW$$

Where *w* = the number of eggs in the sub-sample; *W* = total weight of sub sample and *OW* = total weight of the preserved ovaries of the particular specimen. Relative fecundity was calculated as number of eggs per gram body weight. Accordingly, relationships between fecundity and carapace width/ body weight/egg sac weight were finally obtained.

### Size at 50% Maturity

The following logistic regression model was used to estimate the proportion of mature *P. pelagicus* by carapace width (CW) (King 1995):


$$P=\frac{1}{1+\exp\;(-a\;(CW-b))}$$

Where *P* is the predicted proportion of mature crabs at a particular CW and *a* and *b* are estimated parameters (McCullagh & Nelder 1989), *a* describing the shape of the curve and *b* being the inflection point where 50% of crab for that CW are mature, L_50_. The negative log likelihood (−log_e_L) was calculated as:


$$-Log_{e}L=-[M\;log_{e}P-ILog_{e}(1-P)]$$

Where *M* is the number of mature and *I* is the number of immature *P. pelagicus*. Excel Solver was used to minimise −log_e_L while estimating parameters *a* and *b*. L_50_ value was then estimated by substituting *a* and *b* values in the logistic regression equation.

### Stomach fullness

The dorsal side of the body of the collected specimens of *P. pelagicus* was cut open and the foregut was removed carefully. A total of 613 stomachs were removed and visually observed for stomach-fullness. The removed stomachs were classified according to the degree of fullness as 1, 0.75, 0.5, 0.25 and 0. The percentage occurrence of stomach fullness was determined with respect to two maturity levels (mature/immature).

### Analysis of Types of Foods

The food contained in foreguts were fixed with 10% buffered formalin and stored in 70% ethanol prior to being cut open and their contents transferred into Petri dishes with distilled water. The prominent food components of the gut contents were separated and identified under a compound microscope. Remaining contents were further observed under the light microscope to identify other food items. As is characteristic of brachyurans, most of the food items were found to be unidentifiable as a result of having been highly crushed and hence only the hard structures that could be identified were relied upon for determining food composition and further evaluation. Gut contents were broadly classified into eight categories, as follows:

1. Crustacean remains: Crustacean appendages; body parts of crabs; isopod and stomatopod parts
2. Fish remains: Tissue parts, fins, scales, bones, and vertebrae
3. Molluscan remains: Parts of bivalve and gastropod shells
4. Detritus: Organic matters (decomposed plant materials)
5. Inorganic remains: Sand and mud
6. Synthetic materials: Fish net materials
7. Eggs: Fish and others (crabs and macrocrustaceans)
8. Miscellaneous remains: Filamentous algae, nematodes, polychaetes, and unidentified items

### Estimation of Percentage Frequency of Occurrence

The contents of food in the stomach were considered for analysis and calculation. Immature crabs and mature crabs were sorted according to the appearance of gonad. For each specimen, the whole stomach content was segregated according to food-groups, and each group’s contribution was determined under light microscope. Dominance of food groups were evaluated by ranking them by their percentage frequency of occurrence (Wear & Haddon 1987; Williams 1981) as follows:


$$\begin{array}{l} \text{Frequency of occurrence}=(\text{Number of stomachs with particular food}\\ \text{group}/\text{Total number of stomachs observed})\times 100 \end{array}$$

## RESULTS

### Summary of Biological Data

**Figure 2:**
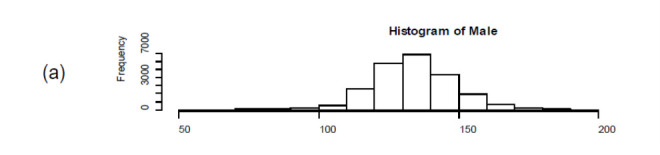

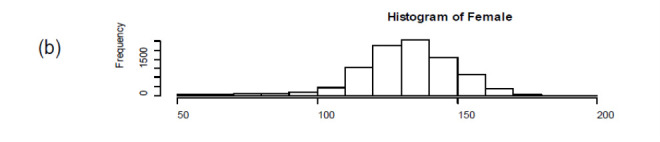

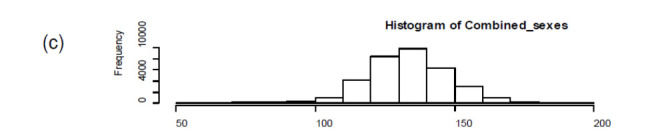
Histogram of Carapace Width (CW) for (a) males (b) females and (c) combined sexes of *P. pelagicus* in Northern coastal waters, Sri Lanka.

The Carapace width of male *P. pelagicus* in Jaffna ranged from 53 mm–196 mm whereas the CW of female *P. pelagicus* ranged from 52 mm–196 mm (Table 1). The average CW of a male *P. pelagicus* in the catch was 133.5 mm (SD = 17.3) whereas the average CW of a female *P. pelagicus* was 135.1 mm (SD = 14.4). As per the Welch Two Sample *t*-test (or unequal variances *t*-test), the CW between males and females were significantly different (*p*-value < 0.001). However, the histograms of males, females and combined sexes were quite similar in shape (Fig. 2).

A considerable monthly variation in the average size of *P. pelagicus* caught in terms of CW could be observed for the study period. The highest average CW (138.1 mm) was observed in September whereas the lowest average CW (130.4 mm) was observed in May. Also, the highest average CW (136.2 mm) was reported in Chatty whereas the lowest average CW (116.8 mm) was reported in Nawanthurai lagoon. The average body weight of a male *P. pelagicus* in the catch was 184.4 g (SD = 67.5) whereas the average body weight of a female *P.pelagicus* was 169.8 g (SD = 62.0).

### Sex Ratio

**Figure 3:**
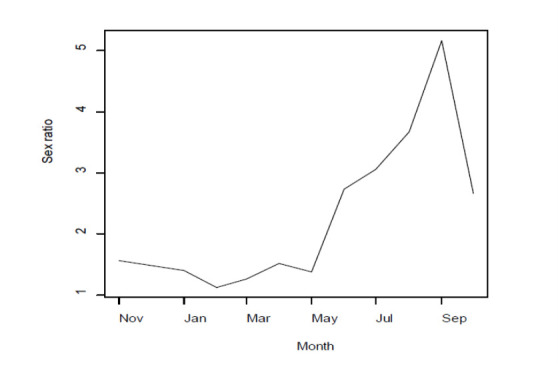
Monthly variation of sex ratio (Male: Female) of *P. pelagicus* in Northern coastal waters, Sri Lanka (November 2014–October 2015).

Based on the biological data of *P. pelagicus* collected at the field for a period of one year (November 2014 to October 2015), the estimated sex ratio between male and female was 1.9:1. However, a considerable seasonal variation could be observed among different months of the year (Fig. 3).

### Size at 50% Maturity

**Figure 4:**
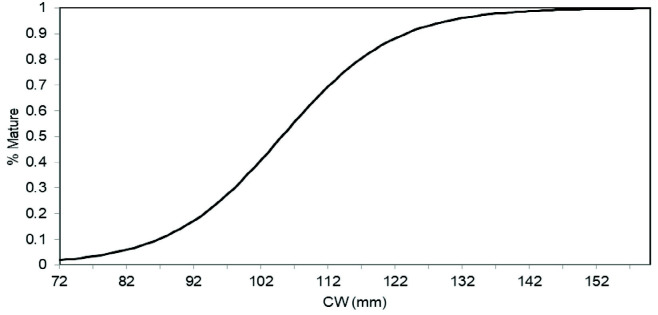

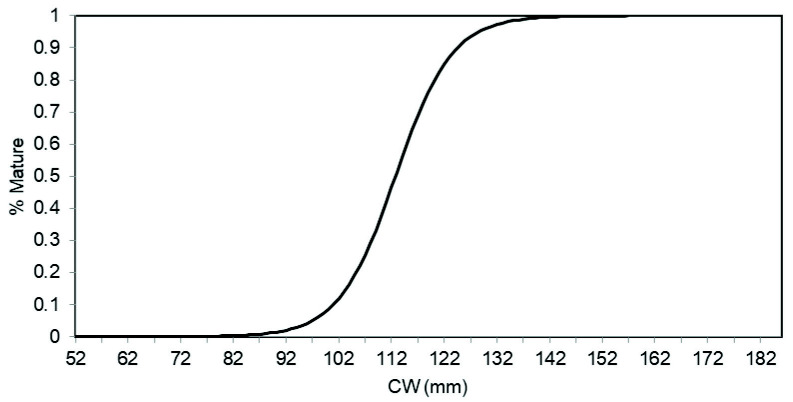
Estimated maturity curves for (a) male and (b) female *P. pelagicus* in Northern coastal waters, Sri Lanka.

The estimated size (CW) at 50% maturity for male and female *P. pelagicus* were 105.1 mm and 112.8 mm, respectively (Fig. 4). *P. pelagicus* represented six maturity stages of female crabs: immature (F1), early maturing (F2), late maturing (F3), gonad mature (F4), berried females (F5), and spent (F6) were identified in the study.

### Spawning Season(s)

**Figure 5:**
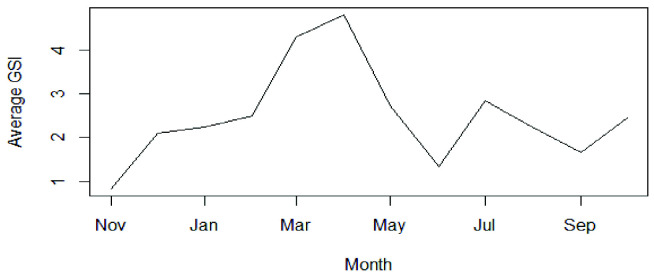
Monthly variation of average Gonado Somatic Index (GSI) in female *P. pelagicus* in Northern coastal waters, Sri Lanka (November 2014–October 2015).

The monthly average of GSI in female *P. pelagicus* ranged from 0.83 to 4.80. The lowest and highest monthly average values for GSI were estimated in November and April, respectively. Accordingly, the spawning peak was observed during March–April (Fig. 5).

**Figure 6:**
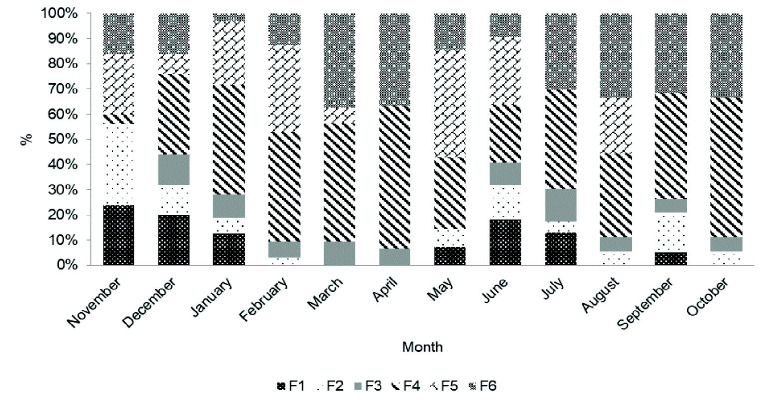
Monthly variation of maturity stages of female *P. pelagicus* in Northern coastal waters, Sri Lanka (November 2014–October 2015).

**Figure 7:**
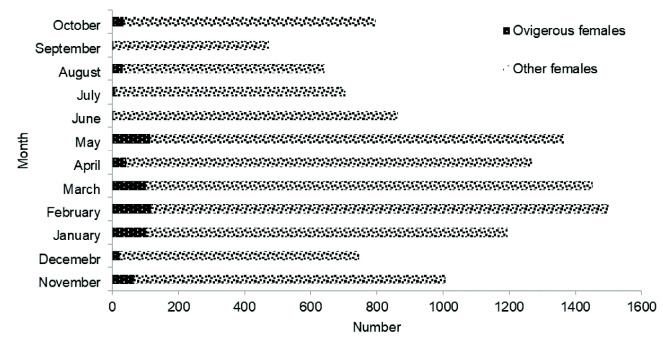
Monthly variation in the present of ovigerous females and other females *P. pelagicus* in Northern coastal waters, Sri Lanka during biological sampling (November 2014–October 2015).

Since late development stages of females were found in each month of the year it could be concluded that *P. pelagicus* breeds thorough out the year (Fig. 6). The biological data obtained at the field sampling was also evidence to show that the ovigerous females were present in the samples throughout the year, but the highest proportion of ovigerous females caught were recorded in February and May (Fig. 7).

### Fecundity

Total fecundity estimated in the study varied from 123,482 to 3,179,928 eggs, with an average of 884,982 ± 676,420 eggs and the relative fecundity was estimated at 3,720 ± 3,003 eggs per gram body weight of a female *P. pelagicus*. The carapace width of the *P. pelagicus* which was used for fecundity estimate varied from 113 mm–177 mm.

Three stages of egg sacs were identified in the study based on their colour; yellow/orange, brown and greyish-black/black. It was noted that yellow/orange colour egg sacs mainly composed of eggs with early developmental stages while the greyish-black colour egg sacs composed of a large number of eggs of late developmental stages.

### Estimated Relationships Between Total Fecundity and Morphometric Parameters

**Figure 8:**
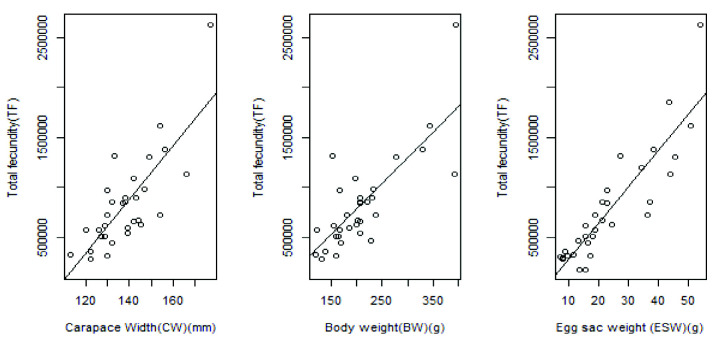
Relationships between some morphometric parameters and total fecundity of *P. pelagicus* in Northern coastal waters, Sri Lanka (Left to right): carapace width (CW) and total fecundity (TF); body weight (BW) and total fecundity (TF); egg sac weight (EW) and total fecundity (TF)

Relationships between total fecundity and morphometric parameters were obtained for *P. pelagicus* (Fig. 8).

The estimated linear relationship between total fecundity (TF) and carapace width (CW) is

*TF =* −*2897951 + 26970CW*
The relationship is significant (*p* < 0.0001, *r^2^* = 0.62).
The estimated linear relationship between TF and Body Weight (BW) is
*TF =* −*252924 + 5178.3BW*
The relationship is also significant (*p* < 0.0001, *r^2^* = 0.62).
The estimated linear relationship between TF and Egg sac weight (EW) is
*TF =* −*80899 + 36335EW*

This relationship is highly correlated than other relationships estimated above (*p* < 0.0001, *r^2^* = 0.8).

### Stomach Fullness

**Figure 9:**
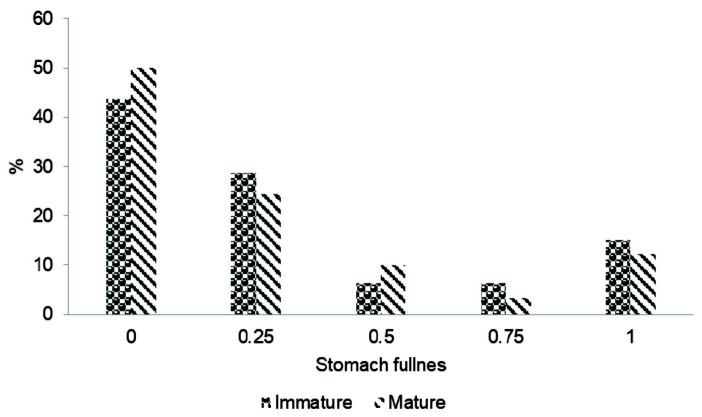
The stomach fullness of mature and immature blue swimming crabs in Northern coastal waters, Sri Lanka.

Out of 613 stomachs observed for stomach-fullness, 533 were mature crabs and 80 were immature crabs. Around 50% of the stomachs of mature crabs were empty whereas around 44% stomachs of immature crabs were empty (Fig. 9).

### Types of Foods

**Figure 10:**
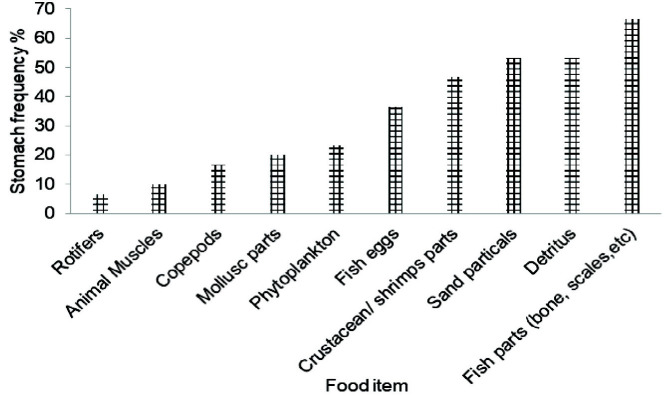
Percentage occurrence of food items in the stomachs of mature blue swimming crabs in Northern coastal waters, Sri Lanka.

The adult blue swimming crab diet comprised of phytoplankton, zooplanktons (copepods and rotifers), fish parts (bone and scales etc.), fish eggs, crustacean/ shrimp parts, mollusc parts, sand particles and detritus. The percentage frequency of occurrence of food items in the adult stomach of *P. pelagicus* was greatly varied: fish parts (bones & scales) = 67%, detritus = 53%, sand particles = 53%, crustacean/ shrimp parts = 47%, eggs (fish and other eggs) = 37%, mollusc parts = 20%, phytoplankton = 23%, copepods = 17%, animal tissue (fish & other tissue parts) = 10% and rotifers = 7% (Fig. 10).

**Figure 11:**
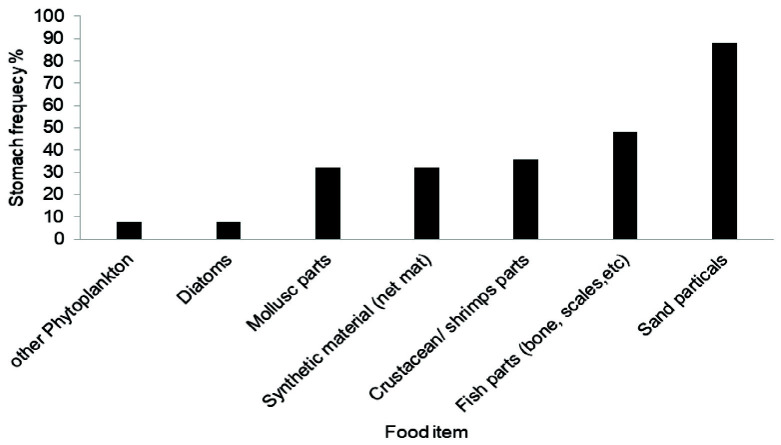
Percentage occurrence of food items in the stomachs of immature blue swimming crabs in Northern coastal waters, Sri Lanka.

The percentage frequency of occurrence of food items in the juvenile *P. pelagicus* were: sand particles = 88%, fish parts (bones and scales) = 48%, crustacean/ shrimp parts = 36%, fishing net materials = 32%, mollusc parts = 32%, phytoplankton = 8%, diatoms = 8% and other phytoplankton = 8% (Fig. 11).

## DISCUSSION

The Palk Bay and Gulf of Mannar are the best-known fishing grounds in the sea for blue swimming crabs in Sri Lanka (Haputhantri *et al*. 2021). Bulk of landings of blue swimming crabs in India has also been reported from the Indian part of Palk Bay and Gulf of Mannar (Josileen & Menon 2007). Still no scientific evidence has been received to confirm that a single blue swimming crab stock is exploited by both Indian and Sri Lankan fishermen. On the other hand, no scientific proof has been received to confirm whether Palk Bay stock is distinct from the Gulf of Mannar stock (SFP 2016). Since a geographic boundary between Palk Bay and Gulf of Mannar exists from a chain of limestone islands covered with sand, as well as sand islands called Adam’s Bridge, two distinct stocks of blue swimming crab could exist in Gulf of Mannar and Palk Bay. The Sri Lankan part of the Palk Bay is known to be the fishing ground of Jaffna, Kilinochchi and Mannar fishermen whereas the Sri Lankan part of the Gulf of Mannar is the fishing ground of Mannar and Puttlam fishermen.

A vast majority of ovigerous females of *P. pelagicus* caught in Palk Bay were reported from January to May, in particular in the months of February and May, and this provides strong evidence that this species spawns in this bay during this period. Moreover, this study reveals that the spawning peak occurs in March/April, likely during the first inter monsoon season. Similar spawning season(s) of *P. pelagicus* has been reported in other areas of the Indian Ocean. The spawning season of *P. pelagicus* in India actively occurred during February–March (Dineshbabu *et al*. 2008). *P. pelagicus* has two peak spawning seasons in East Lampung waters in Indonesia: April–June (main spawning season) and October–November (second spawning peak) (Zairion *et al*. 2015). However, in tropical waters, *P. pelagicus* breeds throughout the year (Batoy *et al*. 1987). Sudtongkong (2006) observed that in *P. pelagicus* in Southern Thailand, all stages of ovarian development occur all year round. The present study also confirms that *P. pelagicus* spawns throughout the year.

The determination of the sex ratio and of the sequence of changes in maturity stages during the year is of considerable importance in building a thorough knowledge of the general biology of an exploited stock (Holden & Raitt 1974). In the present study, there is a significant deviation between the observed and the expected sex ratio between male and female (i.e., 1:1) and this may probably be due to the night time behaviour of males where males become more active than females. This is because they are looking for females that have just shed their shells to mate with. As a result, males become more vulnerable to fishing gears operated during the night time. The sex ratio reported for *P. pelagicus* being relatively closer to 1:1 from November to May than the rest of the period, could probably be attributed to the behaviour of the female crabs during the spawning season. Female crabs become active during this period and they are swimming to release their eggs. Remarkable increase in the sex ratio, in particular during the period from June to September should further be investigated with the factors such as food availability and temperature variation etc. Safaie *et al*. (2012) noted that the deviation of 1:1 sex ratio in male and female *P. segnis* may be due to its behaviour and migration or gear selectivity. Dineshbabu *et al*. (2007) observed that *P. sanguinolentus* (Herbst) from South Karnataka coast, India showed that the annual sex ratio was not significantly different from 1:1. This observation has been made in the trawl fishery. These results are quite possible since the female crabs would be scooped up from the sand by the active trawl fishing gear unlike a passive gear like bottom set gillnets used by Jaffna fishermen for catching blue swimming crab. However, from the point of view of conserving spawning potential, harvesting more males than females is of paramount importance for sustainable harvesting of the fishery resources.

Most crabs reach sexual maturity in 12 months with males growing to a larger size than females (Meagher 1971; Sumpton *et al*. 1994). Maturity stages of female *P. pelagicus* were determined by Dineshbabu *et al*. (2007) and was classified into five categories namely, immature, early maturing, late maturing mature and spent. In this study, maturity stages of females were classified into six categories and the categorisation of ovarian development was mainly based on the size and colour.

In the present study, the length at 50% maturity for male and female blue swimming crab indicates that males mature at a smaller size than females and respective values were 105.1 mm and 112.8 mm. A similar observation has been made from the opposite coasts (East and West) of peninsular Malaysia where the estimated size at 50% for female blue swimming crab (113 mm–118 mm) was higher than for males (105 mm–110 mm) (Asemari *et al*. 2018). The size at first maturity/sexual maturity could vary due to various reasons. Sukumaran and Neelakantan (1996) revealed that the size at first maturity could vary with latitude or location. Moreover, the carapace width at which *P. pelagicus* reaches sexual maturity vary depending on growth rate, which is a direct function of temperature (Dineshbabu *et al*. 2008). De Lestang *et al*. (2003) also revealed that the development of ovarian and egg in *P. pelagicus* is controlled by water temperature. The length at maturity of crabs can be integrated into the management of blue swimming crab fishery in Sri Lanka. Perhaps the most appropriate and practical way of implementing a size regulation appears to be setting the minimum size limits in terms of weight based on the size at 50% maturity of blue swimming crabs. The crab processing companies could be instructed to follow this regulation while they purchase blue swimming crabs for processing. The blue swimming crab fishery is mostly conducted aiming the export market and the prices in the local market are normally much lower than the prices received in the export market.

Knowledge of the fecundity of a species is an important factor in fish stock management (Holden & Rait 1974). Fecundity is used to calculate the reproductive potential of a stock and the survival from egg to describe a fish which is spawning for the first time (Holden & Raitt 1974). Fecundity of crabs like *P. Pelagicus* may vary due to different factors such as age, size, nourishment and ecological conditions of the water body which they live in etc. (Safaie *et al*. 2012). Variation in fecundity is primarily a reflection of variation in the size of the crab at maturity (Arshad *et al*. 2006). As observed in the present study, fecundity of *P. pelagicus* increases with increased carapace width and body weight. Further, the fecundity ranged from 123,482 to 3,179,928 eggs, with an average of 884,982 ± 676,420 eggs. In the West coast of Australia, the fecundity of *P. pelagicus* has ranged from about 78,000 eggs in small crabs (CW=80mm) to about 1,000,000 eggs in large crabs (CW=180 mm) (Kumar *et al*. 2000). In Southern Thailand, the fecundity has ranged from 27,040 to 1,725,179 eggs, with an average of 474,550 eggs (Sudtongkong 2006). Accordingly, it seems that fecundity of *P. pelagicus* in the Northern waters of Sri Lanka is comparatively higher than the fecundity of the same species reported in Western Australia and Southern Thailand. Moreover, the reproductive potential of blue swimming crab in northern waters of Sri Lanka is apparently higher than the reproductive potential of the same species inhabited in the other two countries. Perhaps, a comparative regional study may be needed to be carried out in order to come to a strong conclusion.

*P. pelagicus* is most active in foraging and feeding at dawn and dusk (Sumpton & Williams 2002) or dusk (Grove-Jones 1987; Smith & Sumpton 1987; Wassenberg & Hill 1987). Remarkably higher percentage of empty stomachs observed under the study in both mature and immature crabs could be due to factors such as lack of food availability in the environment and different digestibility rates of food items. Also, crabs stop feeding prior to and during moulting. The difference in stomach fullness between mature and immature crabs was significant (*p* ≤ 0.05).

Blue swimmer crabs feed on animal material such as fish, crustaceans and molluscs, plant materials and debris (Kunsook *et al*. 2014; Josileen 2011; Williams 1981). The results of the present study are also in agreement with the findings of the above studies. Moreover, a considerable difference could be observed in the feeding pattern between immature and mature crabs. Since juveniles and adults are inhabited in two habitats, the abundance and distribution of prey may differ. The higher frequency of occurrence of sand particles and fishing net material in the stomach of immature crabs is evidence for their non-selective food habit nature.

## CONCLUSION

Though a spawning peak of *P. pelagicus* in the northern waters of Sri Lanka was identified in March / April of the year, the presence of ovigerous females throughout the year was evidence for their continuous spawning. The total fecundity of *P. pelagicus* shows significant linear relationship with carapace weight, body weight and egg sac weight but the relationship between the total fecundity and egg sac weight was highly correlated than other relationships. The presence of male crabs in the catch in a higher percentage throughout the study period was attributed to the behaviour of blue swimming crab. The presence of sand particles and fishing net material in the stomach of immature crabs in a higher percentage was evidence for their non-selective food habit nature. The results and findings of the present study could presumably be made use of better management of the capture-based fishery and perhaps for blue swimming crab farming. The study emphasises the importance of introducing minimum size regulations in terms of weight of crabs as a management measure for discouraging the harvest of immature crabs and this could practically be implemented in a regulation that is applicable for sea food processing companies when purchasing crabs for processing.

## Figures and Tables

**Table 1:** Summary of carapace width and body weight of blue swimming crab in Jaffna, Sri Lanka: from November 2014 to October 2015.

Sex	No.	Carapace width (mm)				Weight (g)			

		Min	Max	Ave.	SD	Min	Max	Ave.	SD
Male	23,109	53	196	133.5	17.3	9	573	184.4	67.5
Female	12,014	52	196	135.1	14.4	5	548	169.8	62.0
Combined	35,123	52	196	134.6	15.5	5	573	179.4	67.1

**Plate 1:**
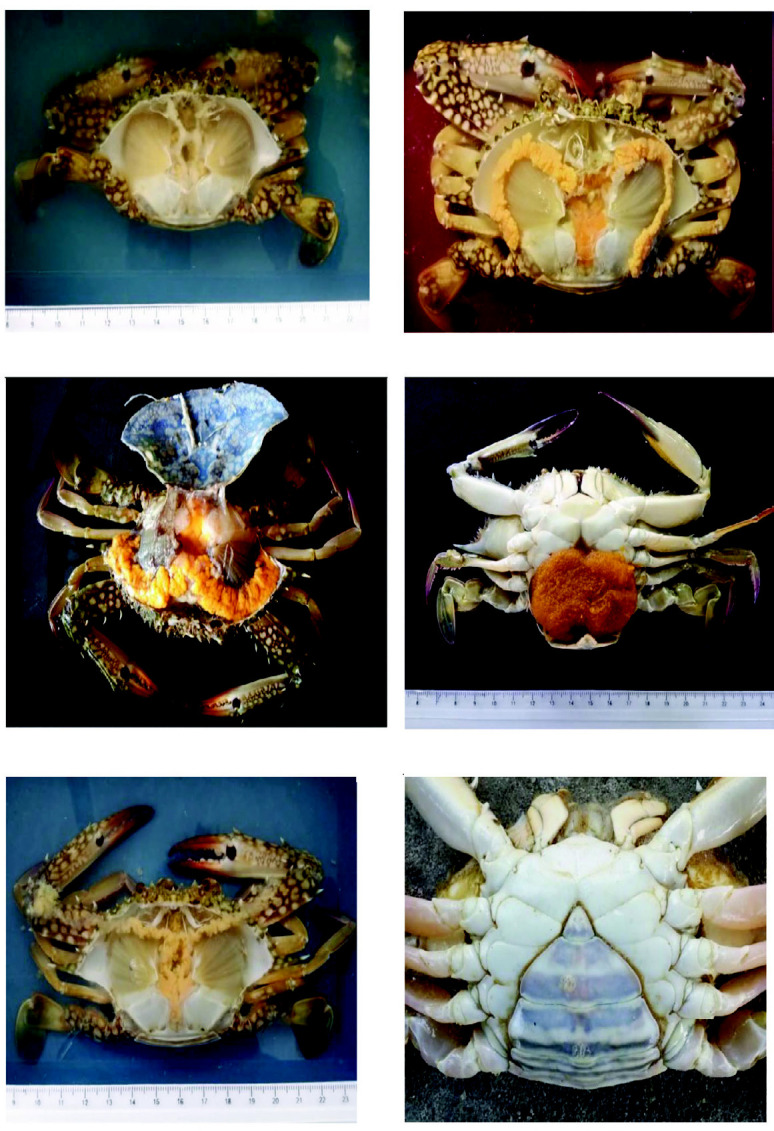
Maturity stages of female blue swimming crab in Jaffna (a) early maturing −F2, (b) late maturing −F3, (c) gonad mature −F4, (d) berried females −F5, (e) spent −F6, (f) abdomen turned into coffee brown colour in the spent females.

**Plate 2:**
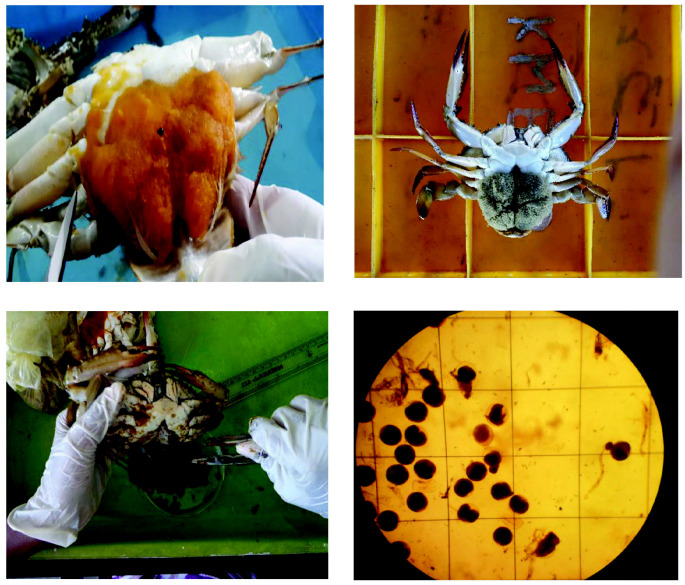
Three stages of blue swimming crab egg sacs identified in the study in Jaffna: (a) yellowish/orange, (b) brown, (c) greyish-black/black and (d) microscopic view of late embryonic stages in greyish-black/black eggs

**Plate 3:**
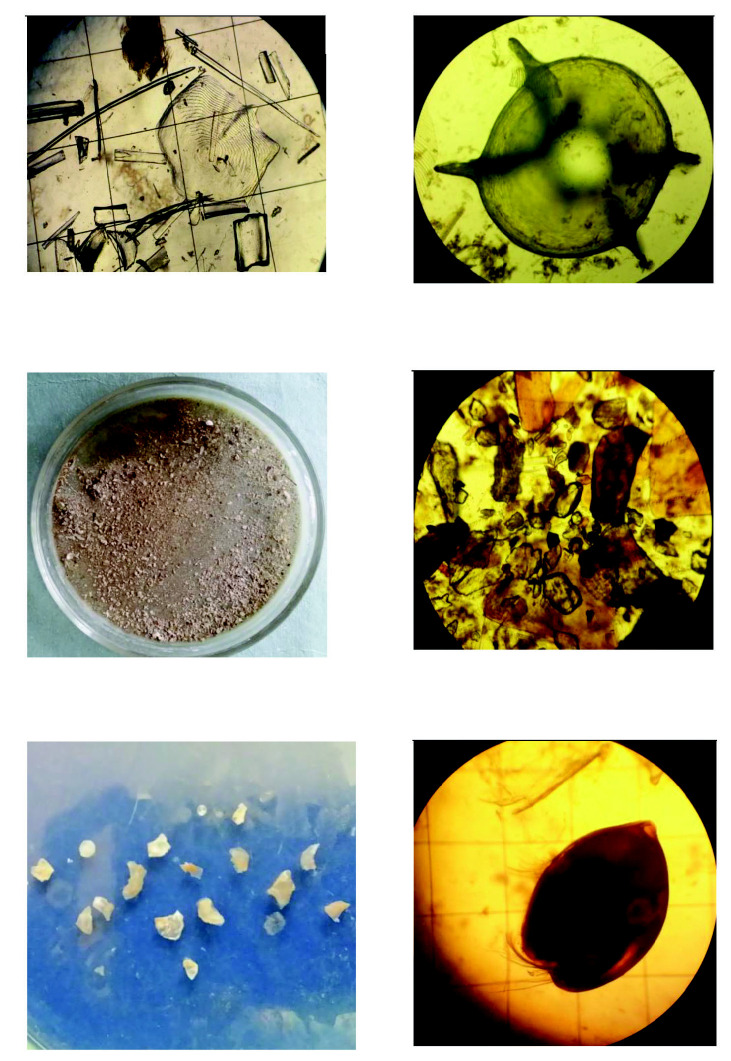

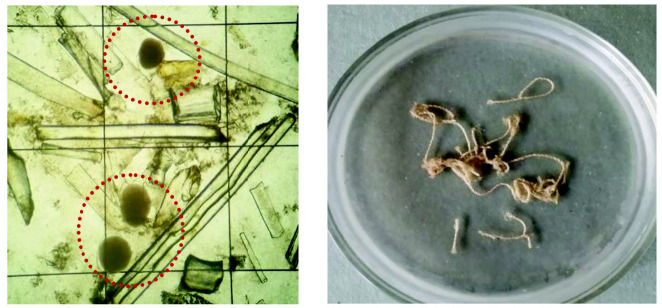
Some food items found in the stomach of blue swimming crab in Jaffna
